# Using Animal Models to Study the Role of the Gut–Brain Axis in Autism

**DOI:** 10.1007/s40474-017-0111-4

**Published:** 2017-05-12

**Authors:** Jess Nithianantharajah, Gayathri K. Balasuriya, Ashley E. Franks, Elisa L. Hill-Yardin

**Affiliations:** 1 Florey Institute of Neuroscience and Mental Health, The University of Melbourne, 30 Royal Parade, Parkville, VIC 3052 Australia; 2 School of Health and Biomedical Sciences, RMIT University, Bundoora, Melbourne, VIC 3083 Australia; 3 Department of Physiology, Anatomy and Microbiology, La Trobe University, Plenty Road, Bundoora, Melbourne, VIC 3086 Australia; 4 Department of Physiology, The University of Melbourne, Corner of Royal Parade and Grattan St, Parkville, VIC 3010 Australia

**Keywords:** Autism mouse models, Cognition, behavioural assays, Gastrointestinal dysfunction, Microbiome, Comorbidities

## Abstract

**Purpose of Review:**

Individuals with autism spectrum disorders (ASD) commonly also suffer from gastrointestinal (GI) dysfunction; however, few animal model studies have systematically examined both ASD and GI dysfunction. In this review, we highlight studies investigating GI dysfunction and alterations in gut microbiota in animal models of ASD with the aim of determining if routinely used microbiology and enteric neurophysiology assays could expand our understanding of the link between the two.

**Recent Findings:**

Gut–brain axis research is expanding, and several ASD models demonstrate GI dysfunction. The integration of well-established assays for detecting GI dysfunction into standard behavioural testing batteries is needed.

**Summary:**

Advances in understanding the role of the gut–brain axis in ASD are emerging; however, we outline standard assays for investigating gut–brain axis function in rodents to strengthen future phenotyping studies. Integrating these findings to the field of animal behaviour is one of the next major challenges in autism research.

## Introduction

Autism spectrum disorder (ASD) is a prevalent neurodevelopmental disorder affecting as many as 1:45 children in the USA [1]. Diagnosis is based on behavioural traits presenting as impaired social communication and repetitive and/or restrictive behaviours [2]. Along with these core diagnostic traits, individuals with ASD experience a range of comorbidities that vary in severity and combination between individuals and negatively impact quality of life. Gastrointestinal (GI) dysfunction is more prevalent in individuals with ASD than in the general population [3], and emerging well-designed clinical studies have identified differences in the microbes that inhabit the GI tract in individuals with ASD compared to controls [4–7]. In this review, we outline the potential for animal studies of ASD to elucidate biological mechanisms associated with gut–brain axis changes. We will focus on two genetic models (the chromodomain helicase DNA binding protein 8 and serotonin transporter (CHD8 and SERT) mouse models, respectively) and two environmental models (valproate (VPA) and maternal inflammation activation (MIA)) of autism because GI abnormalities including altered microbial populations (dysbiosis), in addition to core ASD-relevant behavioural phenotypes, have begun to be investigated in these models. These studies are notable for quantifying GI dysfunction and reporting microbial dysbiosis. Here, we provide an overview of how animal models provide a powerful preclinical tool for understanding biological causes of ASD in the drive to identify targets for new therapies.

## Results

### Use and Validity of Animal Models in ASD Research

The number of animal models of ASD has dramatically increased since the generation of the first genetic mouse model of autism in 2007 [8]; currently, more than 30 models have been reported. Animal models of neurodevelopmental disorders (NDD) are an essential research tool—they allow detailed investigation of associated pathology through the use of powerful but invasive techniques on a background of controlled environmental and genetic factors. Although a single animal model cannot fully replicate the entire disorder state observed clinically, it can, however, model the expression of distinct traits or endophenotypes (including comorbidities) [9] to enable an in-depth analysis of pathological mechanisms and provide potential therapy targets for future clinical applications. In this review, we focus on recent studies assaying gut–brain axis changes in rodent models of ASD (Table 1).

**Table 1:** Gut–brain axis studies in animal models of ASD

Animal model	GI phenotype	Reference
*CHD8* antisense knockdown zebrafish	Reduced enteric neuronal numbers and slow GI transit.	Bernier et al. [10]
*CHD8* heterozygous mice	Shorter GI tract and tendency for slower GI transit	Katayama et al. [11]
SERT G56A	Slower motility, slower GI transit, fewer neurons, reduced villus height and colonic crypt depth, reduced intestinal permeability	Margolis et al. [12••]
Poly(I:C)/MIA mice	Increased intestinal permeability, increased interleukin-6 (IL-6) in gut, altered microbiota, increased levels of 4-EPS (4-ethyl phenylsulphate) in serum, ASD-like behavioural abnormalities in the offspring	Smith et al [13]; Hsiao et al. [14]; Choi et al. [15••]
Prenatal VPA exposure mice	Increased inflammation in gut, brain, altered microbiota, ASD-like behavioural abnormalities in male offspring	de Theije et al. [16]; de Theije et al. [17]; Lucchina and Depino [18]; Kazlauskas et al. [19]

Examples of animal models in which gastrointestinal abnormalities and/or altered microbial populations have recently been identified include genetic models of ASD, environmental models such as maternal inflammation activation (MIA) and exposure to the antiepileptic, valproate (VPA)

In studying underlying biological mechanisms of NDDs, it is important that animal models adhere to criteria regarding face validity (phenotypes demonstrated by the model relevant to patient diagnostic traits, that is, for ASD, impaired social interaction/communication and repetitive and or restrictive interests), construct validity (the method of generation of the model is relevant to pathology, e.g. a genetic mutation identified in individuals with ASD) and predictive validity (rescue of phenotypes by clinically relevant treatments). Numerous standardised behavioural analyses are now well established for rodent models (reviewed in [20••]).

### Assays Relevant to Gut–Brain Axis Function

In addition to the social and communication impairments in ASD, repetitive or restricted behaviours are core to the disorder. Repetitive behaviours or deficits in response control in ASD are thought to be symptoms of dysfunction in executive processing, which impacts other cognitive functions such attention and cognitive flexibility [21, 22].

Modelling some of the complex cognitive and executive processes often assessed in the clinic, novel behavioural tools such as the rodent touch screen cognitive tests have emerged. This technology allows a systematic and comprehensive analysis of the cognitive profile of rodent models of a NDD within a single testing environment [23, 24]. Moreover, different cognitive domains relevant to the disorder such as mental flexibility, response control and attention can be measured, with strong translational implications [25, 26].

The role of the central nervous system (CNS) in behaviour is well studied in animal models of ASD; however, the involvement of microbes and GI function is a new and rapidly emerging area of research. The complex enteric nervous system (ENS) of the GI tract, also known as the ‘second brain’, contains roughly equivalent numbers of neurons as the spinal cord. Interactions between the CNS and GI tract occur via multiple neural and endocrine pathways. Predominant neural pathways include the peripheral nervous system (e.g. the vagal nerve, sympathetic nervous system and ENS). The microbiota residing in the GI lumen also impact CNS function; however, the precise pathways involved are not well defined. The ENS is required for GI motility and secretion and comprises two neuronal plexuses adjacent to longitudinal and circular muscle layers in close proximity to microbes in the gut lumen (see [27•] for review). There is also significant cross talk between the immune system, the microbiota and the nervous system which impact behaviour. The ENS regulates GI function and is located in close proximity to microbial populations in the lumen of the GI tract. Gut microbes produce metabolites that function as neurotransmitters and have been established to modulate mood and behaviour; however, our understanding of the precise pathways involved is in the early stages [28, 29]. For example, recent findings from animal models demonstrate that gut microbes regulate serotonin levels and in turn mood and behaviour via their influence on microglial cells in the CNS [14, 30••], but the role of the ENS in this process is only beginning to be understood.

Multiple approaches for assaying gut function are routinely used in enteric neuroscience research and can be incorporated into phenotyping studies of ASD animal models. These also have the potential to be combined with microbial and cognitive behavioural assays for a multidisciplinary holistic view across the major components driving interactions. This multidisciplinary approach will be crucial for improving our understanding of the complex systems physiology underlying behavioural impairments in ASD and the development of novel therapies. The gut houses the largest immune system in the body, and the immune and nervous systems are in constant bidirectional communication [13, 15••, 31–35]. Gut microbiota interact closely with neuroimmune pathways [13, 31–34, 27••, 28••, 29••] and play an important role in educating the developing immune system [28, 36•, 37]. Gut microbial populations are additionally modified by environmental factors such as diet [37–39] and stress [29], and these are areas that are currently actively being researched in ASD models.

Recent advances in sequencing technology and bioinformatic analysis have exponentially increased the understanding of the microbiome in animal models. Initial studies of the microbiome began through culturing of individual, primarily pathogenic, organisms [40]. High-throughput sequencing now allows the cataloguing of entire microbiomes on different hierarchical levels including chromosomal DNA (genomic), transcribed RNA (transcriptomic) and protein production (proteomic) levels. A combination of techniques allows a holistic approach of the microbial community function and how the microbiome itself may have multifaceted interactions within the gut and brain. Microbiome studies must take into account a number of environmental factors (e.g. home cage housing, diet, age of weaning) as well as ensuring that they are designed to allow sufficient statistical power to provide robust outcomes. High-throughput techniques need to be complemented by traditional culturing, where possible, and temporal and spatial analysis of microbes of interest as well as the application of ecological theory to the complex and continuously evolving microbial community.

**Fig. 1:**
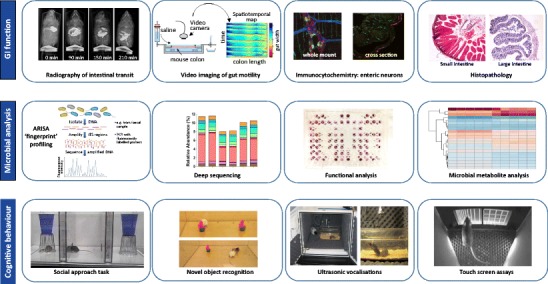
Assays for investigating gut–brain axis function in rodents. A range of established assays are available for studying gut–brain axis function in rodent models. Functional and structural assays for determining impairments in gastrointestinal function (*top*) include in vivo serial X-ray imaging to investigate intestinal transit after oral barium sulphate gavage and ex vivo video imaging to quantify changes in GI motility [42, 44]. Using this method, propagating contractions in a physiological organ bath are video recorded and converted to spatiotemporal maps for detailed analysis [41]. Immunofluorescent labelling of the ENS in whole mount and cross-sectional intestinal preparations and histological labelling for assessing intestinal integrity and morphology (e.g. [45]) can also be undertaken. Structural, functional and predictive approaches to characterise changes in microbiota are also available (*middle*). ARISA fingerprints are used to examine the microbial community structure of the intestine, and deep sequencing allows identification of altered abundance in different microbial species. Functional analyses can be carried out by studying Community-Level Physiological Profiles (CLPP) utilising colorimetric assays. Microbial metabolite analysis is carried out by extracting faecal material and analysis through Gas Chromatography–Mass Spectrometry (GC-MS) methods. Cognitive behavioural analyses (*bottom*) should employ a battery of assays such as the social approach [20••, 49, 52], novel object recognition [50, 54] ultrasonic vocalisation assays [51, 55, 56]. and touchscreen tests [25, 26, 57] which provide a robust method to assess cognitive abilities that are clinically relevant

In this review, we highlight established assays in the fields of enteric neurophysiology [12, 41–45], microbiology [46–48] and rodent cognitive behaviour [20••, 23–26, 49–54] that in combination will expand our knowledge of gut–brain axis function in animal models of ASD (Fig. 1).

### Gut–Brain Axis Studies in Rodent Models of ASD

ASD aetiology is complex and likely to be the due to a combination of genetic and environmental factors in many cases. Therefore, we review pertinent studies of two genetic and two environmental models of ASD in which GI dysfunction and/or microbial dysbiosis is present in order to highlight the use of techniques useful for identifying aberrant gut–brain axis function in animal models in this field. Recent findings suggest ASD-associated mutations in two genes; the chromatin remodelling gene, CHD8, and the solute carrier family 6 member 4 gene (*Slc6a4*) encoding the serotonin transporter (SERT) protein confer GI dysfunction in animal models [10, 11, 12••]. Similarly, studies using the maternal inflammation activation (MIA) model (e.g. [13, 14, 15••]) and the administration of the antiepileptic, valproate (VPA) [16, 17], show altered microbial populations, behavioural changes and potential therapeutic targets through the analysis of components of the gut–brain axis.

#### CHD8 Mutations Are Associated with GI Dysfunction

Mutations in the chromatin-remodelling gene CHD8 increase susceptibility to ASD and result in macrocephaly, facial phenotypes and GI issues in affected individuals [10]. Bernier and colleagues re-sequenced the CHD8 gene in 3730 children with developmental delay or ASD and identified 15 mutations in CHD8 that were considered potentially disruptive [10]. Previously, these authors identified nine de novo mutations in 2446 ASD patients, all impacting normal protein expression [58]. Importantly, recontacting families involved in this study enabled detailed phenotypic data for individuals with CHD8 mutations. Of the 15 children expressing CHD8 mutations, 12 (80%) had GI problems. Of these, 60% reported recurrent and consistent constipation, which was often reported as periods of constipation followed by loose stool or diarrhoea. The rate of constipation in the CHD8 cohort (15 children) was higher when compared to children diagnosed with ASD but negative for the CHD8 mutation [10] suggesting involvement of the CHD8 gene in GI dysfunction.

To determine the effects of mutating CHD8 on GI function, Bernier and colleagues assessed dose-dependent effects of CHD8 mutations using a knockdown approach in zebrafish. They found that 40–60% of embryos injected with antisense probes (or morpholinos, a molecular tool to inhibit gene expression and prevent protein synthesis) had fewer enteric neurons than controls as identified by fluorescent immunocytochemical labelling with the pan-neuronal marker, Hu. At increasing concentrations of morpholinos, the effect on enteric neuronal number was more severe [10]. On average, the number of enteric neurons in the hindgut was reduced by an astounding 50% following knockdown and this was exacerbated in a dose-dependent manner suggesting a major role of CHD8 in neuronal development within the GI tract. These findings were a catalyst for further phenotypic analysis in mice lacking CHD8 in order to advance the understanding of disease mechanisms.

Recently, Katayama and colleagues examined mice heterozygous for mutations in CHD8 [11]. The CHD8 gene produces two isoforms of the CHD8 protein: one the full length and the other containing only the N-terminal domain. Mice homozygous for mutations in either of these CHD8 isoforms are embryonic lethal [59, 60]. In contrast, CHD8 heterozygous (het) mice are viable and show increased anxiety, abnormal sensorimotor arousal and gating (as tested by prepulse inhibition paradigms), altered social interaction in some parameters and increased brain weight [11]. CHD8 het mice had neurodevelopmental delay as identified using mouse brain gene set enrichment analysis and suppression of many neuronal genes via the RE-1 silencing transcription (REST) factor. Somewhat surprisingly, learning and memory tests did not show major differences in CHD8 het mice compared with controls. Mutant mice, however, had an increased perseverative phenotype (relevant to ASD behavioural traits) following assessment using the T-maze left-right discrimination test [11]. Katayama and colleagues also reported fewer social contacts and reduced total duration of active contacts (sniffing and following behaviour). In contrast, another group found that social behaviour in CHD8 het mice was unchanged compared to wild-type littermates [61]. Although environmental differences (potentially involving microbial–behavioural interactions) may contribute to disparate behavioural results in this model, in line with observations of GI dysfunction in those with ASD, Katayama et al. observed that the intestine length in mutant mice was reduced and intestinal transit in 9-week-old mice tended to be slower [11].

Relevant to observations in rodent and zebrafish models, mutations in the CHD8 equivalent homologous gene in drosophila (*kismet*) result in displaced expression of the hedgehog (*hh*) gene and alter the development of the central drosophila wing region [62]. Given the strong relevance of CHD8 to ASD relevant behaviour and morphology, the analysis of GI function (and other ASD-relevant phenotypes) in drosophila expressing mutations in *kismet* could be informative for the mechanistic understanding of GI dysfunction in individuals with ASD.

Further information regarding underlying biological causes of GI impairment in the CHD8 children with ASD could be extracted from functional and structural analyses of the GI tract in animal models (e.g. by assessing the number of total enteric neurons and proportions of neuronal subtypes in the myenteric or submucosal plexus). Therefore, the application of neurophysiological GI assays, microbial analyses and clinically relevant cognitive tests to CHD8 mutant models (see Fig. 1) could yield mechanistic findings to improve our understanding of biological mechanisms underpinning GI dysfunction in individuals with ASD.

#### Altered Serotonin Transporter Activity Impairs GI Function

Platelet serotonin levels are increased in one third of individuals diagnosed with ASD [63, 64], and serotonin is primarily GI-derived [65]. Platelets take up serotonin [66] as they circulate through the gut via the serotonin transporter, SERT (encoded by the *Slc6a4* gene). Many hyperactive mutations in SERT have been reported in individuals with ASD, the most common being SERT Ala56 [67] where a glycine residue is converted to an alanine at position 56 of the protein. This transporter is expressed by enterocytes and serotonergic neurons in the ENS. Serotonin is important in development and adult life and is expressed in a large population of cells located in the GI tract including mucosal enterochromaffin cells, mast cells and a subpopulation of enteric neurons. Serotonin plays an important role in regulating GI motility and secretion, and therefore, changes in serotonergic pathways are highly likely to affect GI function. Serotonin is additionally involved in transmitting signals of noxious stimuli, discomfort and pain to the CNS as well as being a potent modulator of inflammatory pathways [5, 30••, 68, 69].

Margolis et al. conducted a thorough study investigating GI function in mice expressing the SERT Ala56 missense mutation (SERT G56A) using well-established neurophysiological and histological techniques [12••]. In order to understand the role of the SERT transporter in GI function, Margolis and colleagues quantified neuronal numbers in myenteric and submucosal plexuses of the colon and assessed mucosal histology and in vivo transit time as well as ex vivo motility using a well-characterised video-imaging technique [41]. This group studied total neuronal number as well as changes in subpopulations of neurons expressing neurochemical markers such as tyrosine hydroxylase (TH), calcitonin gene-related peptide (CGRP) and gamma-amino butyric acid (GABA). In both the myenteric and submucosal plexuses, neuronal numbers for each of these populations were reduced in SERT G56A mutants. Motility patterns analysed in colonic segments freshly dissected from adult mutant mice showed reduced activity as a result of the SERT G56A mutation. Specifically, the frequency, velocity and contraction length of colonic migrating motility complexes (CMMCs; spontaneous, coordinated contractile activity which propagates from the oral to the anal region of the colon [43]) were reduced in mutant mice. Following examination of the small intestinal histological structure in these mice, a reduction in villus height and crypt depth in SERT G56A mice was identified. Furthermore, in the colon of SERT G56A mice, crypt depth was reduced and intestinal permeability was increased [12••]. This study exemplifies the expansion of preclinical knowledge regarding GI dysfunction in ASD mouse models obtained through the use of functional and histological neurophysiology.

#### Maternal Immune Activation Model of Neurodevelopmental Disorders

Mice born to dams treated with the immunostimulant polyinosinic/polycytidylic acid (poly I:C) during gestation to mimic viral infection demonstrate impairments in communication, stereotypic behaviours, anxiety and sensorimotor abnormalities relevant to ASD and dysbiosis of faecal microbiota [14]. Similar to the VPA model discussed below, the imbalance of microbes in offspring of MIA-treated mice was mainly driven by changes in *Clostridia* and *Bacteroidia* bacterial classes. Changes in *Lachnospiraceae* and *Ruminococcaceae* (of the order *Clostridiales*) reflect similar findings in ASD individuals with increased faecal *Clostridium* species [70–72]. Serum levels of adhesion proteins (including tight junction proteins TJP1 and 2 and claudin-8 and claudin-15) were also altered in treated pups suggesting changes in GI mucosal permeability. Interestingly, treatment with *Bacteroides fragilis* both corrected these adhesion protein deficits and improved several ASD-like behaviours in this mouse model.

In addition to the previous studies, other reports have demonstrated that MIA affects foetal brain development and behaviour via changes in cytokine pathways involving IL-6 [13] and interleukin-17a [15••]. These studies, although not focused on functional assays for GI motility, expand our understanding of how microbiota can potentially impact gut–brain axis function in ASD.

#### Altered Microbiota in Environmental Rodent Models of ASD

Prenatal exposure to the anticonvulsant valproate (VPA) is a risk factor for ASD. Exposure to VPA in rodent models results in behavioural impairments (for a review, see [9]). Rodents born following gestational exposure to VPA demonstrate a range of behavioural impairments including decreased social behaviours and lower exploratory activity combined with repetitive/stereotypic-like hyperactivity (e.g. see [73]). VPA exposure also causes decreased ultrasonic vocalisation and sociability in mouse pups in addition to elevated digging and grooming behaviours [74]. More recently, reports of dysbiosis, altered GI morphology and CNS inflammation following prenatal VPA treatment in mice have emerged [16–19].

Specifically, de Theije and others showed that VPA-treated offspring aged 28 days had decreased abundance of *Bacteroidetes* phyla mainly consisting of *Bacteroidales* and increased *Firmicutes* microbial taxa, mainly consisting of *Clostridiales* [16]. When caecal concentrations of short-chain fatty acids were measured, these authors observed increased butyric acid in VPA-treated male mice compared to controls (pups born to dams not exposed to VPA). Changes in the abundance of microbes belonging to these bacterial groups have also been reported in individuals with ASD [5, 6, 70, 75] (also see [76••]). The short-chain fatty acids (i.e. acetate, butyrate and propionate) are neuroactive microbial metabolites that cross the blood–brain barrier; therefore, the observed increase in butyric acid in this study may have effects on brain function and behaviour. Of particular relevance to GI dysfunction, male pups (but not females) born to VPA-treated dams also showed epithelial cell loss and neutrophil infiltration in the jejunum and ileum as well as reduced serotonin levels in the GI tract [17]. Although the underlying cause of the sex-specific effects is unknown, a role for VPA in inhibiting testosterone to oestradiol conversion [77] has been proposed which could partly explain this finding.

Recently, the detailed discussion of optimal experimental design in studying environmental effects influencing host–microbe interactions in animal models has emerged [78]. This is important because a low level of reproducibility for behavioural approaches has been identified [79], which slows research progress. Furthermore, issues with reproducibility could be due to subtle methodological differences that alter contributions of microbial populations to behavioural outcomes. The use of these best practice guidelines together with validated assays should enable greater consistency between laboratories when endophenotyping animal models of ASD.

## Conclusions

Alterations in gut–brain axis signalling affect mood and behaviour; however, current understanding of the precise biological signalling pathways involved is incomplete. As summarised in this review, the use of genetic and environmental animal models has begun to contribute to characterising biological mechanisms (including a potential role for dysbiosis) underlying these changes. These studies will be important for not only aiding our understanding of basic mechanisms, but also assisting in identifying future therapeutic targets to improve outcomes for those with ASD. However, in light of the range of techniques outlined here that are routinely available, further exploration of functional and structural GI changes in animal models with detailed histological analysis of ENS morphology and mucosal integrity is required. In addition, emerging data suggest that modulating gut microbes impact behaviour and cognition; therefore, future research in animal models using appropriate behavioural experimental assays is required to gain a more comprehensive understanding of the role of the gut–brain axis in the context of ASD.
